# *Samaki Salama* – Promoting healthy child growth and sustainable fisheries in coastal Kenya: A study protocol

**DOI:** 10.3389/fpubh.2022.934806

**Published:** 2022-10-21

**Authors:** Ivy Blackmore, Andrew Wamukota, Elizabeth Kamau-Mbuthia, Austin Humphries, Carolyn Lesorogol, Rachel Cohn, Catherine Sarange, Francis Mbogholi, Clay Obata, Christopher Cheupe, Joaquim Cheupe, Lisa Sherburne, Melissa Chapnick, Mary Kate Cartmill, Lora L. Iannotti

**Affiliations:** ^1^Brown School, Washington University in St. Louis, St. Louis, MO, United States; ^2^Department of Environmental Sciences, Pwani University, Kilifi, Kenya; ^3^Department of Human Nutrition, Egerton University, Nakuru, Kenya; ^4^Department of Fisheries, Animal and Veterinary Sciences, University of Rhode Island, Kingston, RI, United States; ^5^Graduate School of Oceanography, University of Rhode Island, Narragansett, RI, United States; ^6^USAID Advancing Nutrition, JSI Research & Training Institute, Inc, Arlington, VA, United States; ^7^Department of Nutrition and Health Sciences, Emory University, Atlanta, GA, United States

**Keywords:** stunting, nutrition, food security, sustainability, fisheries, social marketing

## Abstract

**Background:**

One in five young children globally suffer the consequences of stunted growth and development and millions experience deficiencies in zinc, iron, iodine, vitamins A and B12, nutrients found bioavailable in fish foods. Small-scale fisheries have the potential to generate income and augment fish consumption while being environmentally sustainable if appropriately managed. However, those engaged in small-scale fisheries are often marginalized, poor, and malnourished. The *Samaki Salama* project seeks to better understand and address these challenges through a three-arm, longitudinal matched cluster study which evaluates the impact of an integrated nutrition social marketing and modified fishing trap intervention.

**Methods:**

There will be 400 small-scale fisher households enrolled from Kilifi County, Kenya and residing in communities matched on location (rural), livelihoods, and child nutritional status. The sample will include mothers and other caregivers, children 6–60 months, and fishers in the family. Applying a cluster design, the matched communities will be divided into three groups: (1) control (*n* = 200); (2) multi-component nutrition social marketing intervention to fishers, mothers, and health workers (*n* = 100); and (3) multi-component nutrition social marketing intervention plus modified fishing traps and training (*n* = 100). Primary outcomes include child growth, fish food intakes, and fisheries yield of mature fish. Secondary outcomes are diet diversity, child diarrheal morbidity, and fisheries revenue. A process evaluation will be used to monitor and ensure fidelity of intervention delivery.

**Discussion:**

This study builds on a growing body of literature illustrating the effectiveness of nutrition focused social marketing campaigns to promote active engagement of participants, high compliance to the intervention, and sustained behavior change. The second intervention element of modified fishing traps that allow immature fish to escape enables participants to act on the messaging they receive and promotes sustainable fishing through increased harvest efficiency and reduced catch of immature fish. The integrated approach of the *Samaki Salama* intervention provides an example of how to leverage multiple disciplines to address key challenges to human and environmental health and illustrates a pathway for scaling study innovations to other small-scale fisheries systems.

**Trial registration:**

https://clinicaltrials.gov (NCT05254444).

## Introduction

Human populations continue to experience nutrient deficiencies with dire health consequences associated with diminishing dietary diversity and a lack of access to certain foods. Stunted growth and development arise from these deficiencies and act synergistically with poverty to reduce the potential of societies to achieve productivity and human wellbeing. Globally, the nutrient deficiencies found widely prevalent – zinc, iron, iodine, vitamins A and B12, among others – are also those found bioavailable in fish foods (1, 2). This speaks to the need to ensure access and more equitable distribution of nutrient-dense foods such as fish across populations. Economic, social, and environmental factors act in tandem to influence access and associated nutrition security. Here we propose a study to test the effects of a multi-component nutrition-sensitive intervention to improve nutrition security along with fisheries sustainability and economic resilience in the first 1,000 days of life and beyond.

Definitions of food security commonly include access, availability, and utilization of nutritious and safe diets year-round (3). Most evidence for nutrition interventions, however, comes from trials testing nutrition-specific interventions or those with direct effects on nutrition such as micronutrient supplementation or fortified foods (4). Consequently, there is a need to build the evidence-base for nutrition-sensitive interventions that target food production and income pathways. Our project embeds nutrition objectives and activities in an intervention to increase sustainable fish production through the provision of modified fishing gear, improve household income, and increase awareness about the importance of fish for maternal and child nutrition. We specifically target small-scale fisher households who are particularly vulnerable to food insecurity and malnutrition and lack the necessary extension support and inputs to increase efficient, sustainable fish production.

Poverty and malnutrition disproportionately affect populations engaged in small-scale food production through fisheries, subsistence agriculture, or livestock production (5). Yet, small-scale production provides the majority of food in developing countries and supports the livelihoods of over 60% of the workforce in this sector (6). Of the small-scale food production sectors, fisheries are a major contributor with more than 50 million men and women employed in the sector (7). However, small-scale fisheries (SSF) are underrepresented in the dialogue around the Blue Economy and have been marginalized by large industrial fisheries, aquaculture ventures serving global markets, establishment of no-take fisheries closures to meet conservation goals, coastal development, foreign tourism, and mineral extraction (8–10). Furthermore, considerations of food and nutrition security have been largely absent from Blue Growth initiatives. Our study positions food supply and nutrition security at the forefront of promoting sustainable SSF systems in Kenya's Blue Economy initiatives through a multifaceted intervention that can be scaled to other systems, including aquaculture.

Food production often comes at the cost of the environment and biodiversity (11–13). Compared with industrial fisheries that tend to target a single species, SSF can be more sustainable and achieve greater outputs per unit effort through the utilization of more passive gear types (traps, gill nets, long lines, etc.) that minimize damage to local ecosystems (14). Small-scale fishers are also more likely to keep and utilize a wide range of the marine species that are caught (14). A more diverse catch spreads out the impact of fishing and reduces the risk of a single species collapse or extinction. However, in contexts where SSF have limited resources and gear options, which reduces the types and number of species fishers are able to catch, the level of fishing effort on local marine ecosystems can become unsustainable and stocks may collapse with concurrent harm to the environment (14–16). The potential for over-exploitation and biodiversity loss highlights the importance integrating marine ecosystem sustainability into interventions targeting small-scale fisher households.

Small-scale fisheries in Kenya are chronically overexploited and fish stocks are dwindling as evidenced by a four-fold decrease in marine catch since the 1980s (17). Efforts to combat declines in SSF production in Kenya have focused on improving the management of fishery resources through the establishment of Beach Management Units (BMU), gear restrictions (e.g., spear gun, beach seine) and no-take fisheries closures (18, 19). The *Samaki Salama* project continues efforts to improve fisheries production in coastal Kenya while also integrating critical dimensions of livelihood sustainability and nutrition security. *Samaki salama* means “fish security” in Kiswahili, the local language, and is used as the project name to highlight the economic, ecological, and nutritional facets of the intervention.

### Logic framework

**Figure 1:**
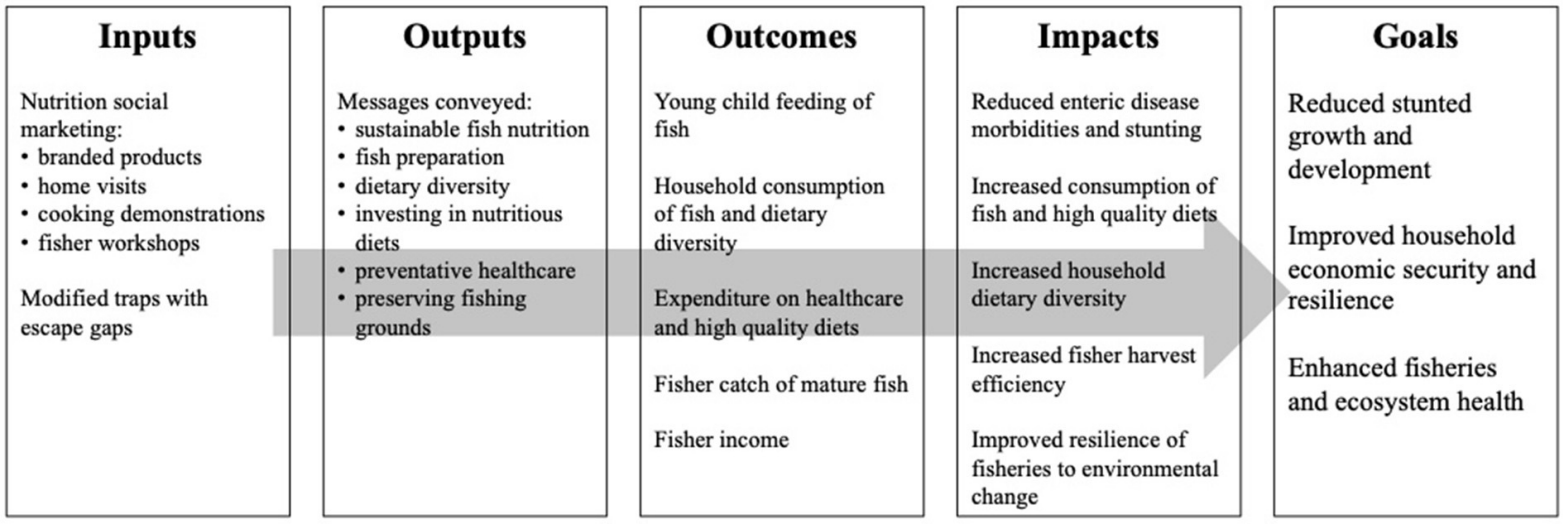
Logical framework.

Figure 1 illustrates the logical framework for our integrated nutrition and fisheries intervention We expect our nutrition social marketing campaign to increase consumption of fish among children under 5 and dietary diversity for households, leading to reduced enteric disease morbidities and reduced stunted growth and development. We anticipate modified fishing traps with escape gaps (to allow immature fish to leave the trap while retaining mature fish) to promote fisheries sustainability and increase fisher income, leading to greater harvest efficiency, improved resilience to environmental change, dietary diversification, and improved nutritional status through both direct consumption and indirect poverty reduction pathways.

## Methods and analysis

### Design

We aim to test the effectiveness of the multi-component *Samaki Salama* intervention as it intersects with nutrition security and fisheries sustainability in Kilifi County, Kenya. Table 1 details the specific aims and hypotheses.

**Table 1:** Study aims and hypotheses.

**Study aims**	**Hypotheses**
1. Determine the effects of a multi-component social marketing campaign to promote fish nutrition, dietary diversity, and food safety on child growth	**Primary** Hypothesis 1: Children in combined intervention groups have increased height-for-age *Z* by 0.2 compared to children in the control Hypothesis 2: Children in combined intervention groups have increased weight-for-age *Z* by 0.10 compared to children in the control Hypothesis 3: Children in combined intervention groups have increased fish food intakes by 100 g compared to children in the control
	**Secondary** Hypothesis 4: Children in combined intervention groups have increased dietary diversity by 1.2 compared to children in the control Hypothesis 5: Children in combined intervention groups have reduced diarrheal morbidity by 5 percentage points compared to children in the control
	**Exploratory** Hypothesis 6: Children in the social marketing + traps intervention group have increased dietary diversity and fish intake compared to children in the social marketing only group
2. Measure the impact of modified fishing traps with escape gaps on catch dynamics and earnings	**Primary** Hypothesis 7: Fishers in the social marketing + traps intervention group have significantly increased fisheries yields of mature fish compared to fishers in the control
	**Secondary** Hypothesis 8: Fishers in the social marketing + traps intervention group will have significantly increased earnings compared to fishers in the control

**Figure 2:**
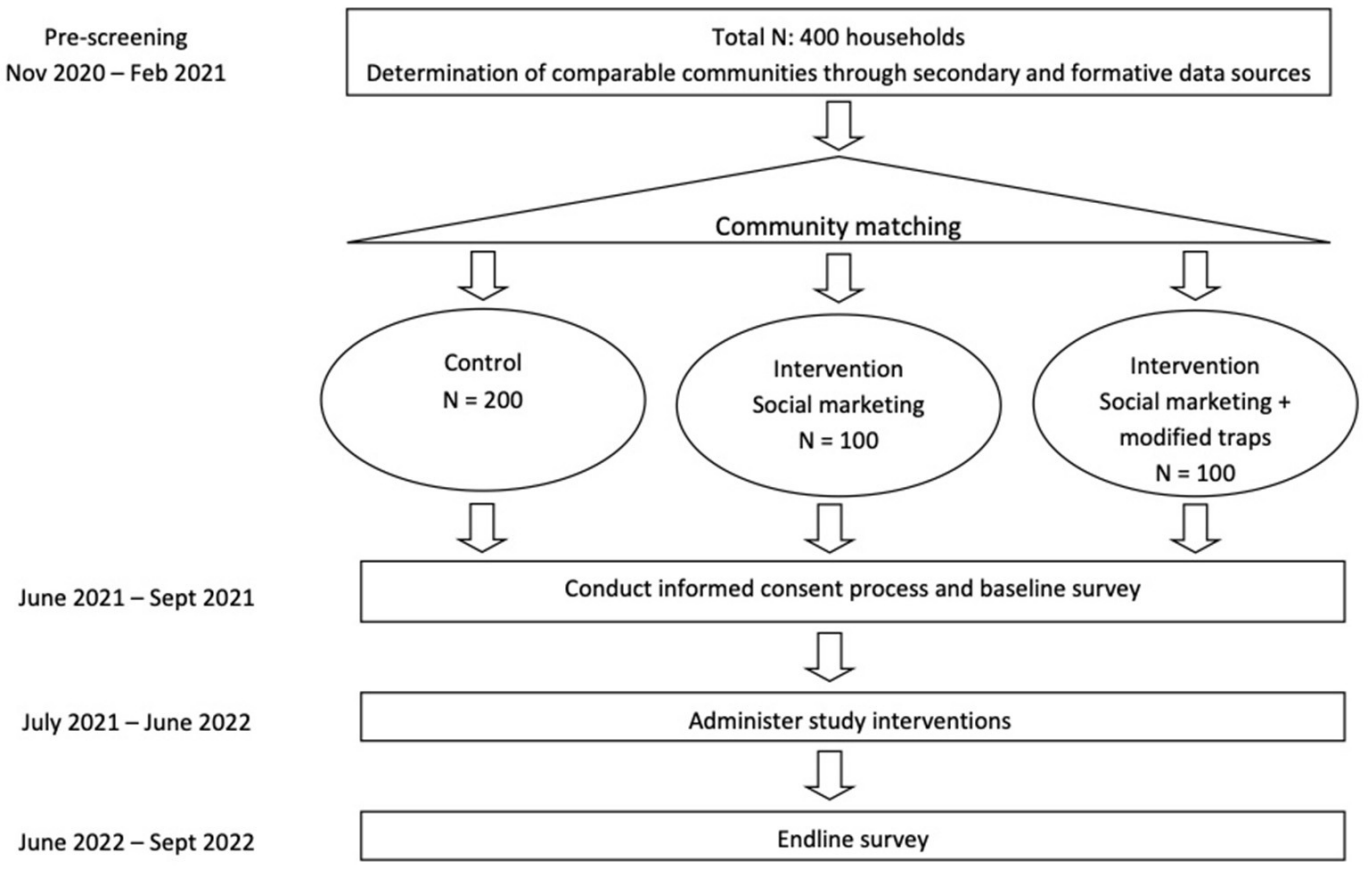
Study design.

The intervention will target communities matched based on location (rural), livelihoods, and child nutritional status. The matched sample will be divided into three groups: (1) control (*n* = 200); (2) multi-component nutrition social marketing intervention to fishers, mothers, and health workers (*n* = 100); (3) multi-component nutrition social marketing intervention plus modified fishing traps with escape gaps and training (*n* = 100). Although our original design was to have two comparison groups with equal numbers, formative research showed insufficient numbers of fishers currently using traps that could be recruited to use modified traps. Further, budgetary constraints precluded distributing traps to all 200 in the intervention group. Thus, we split the intervention group enabling us to test the added effects of modified traps together with social marketing. Figure 2 provides an overview of the study design and implementation process.

Both process and impact evaluations will be carried out using mixed methods. Primary and secondary nutrition outcomes include longitudinal difference-in-difference analyses of parameters – height-for-age *Z* score, stunting prevalence, child fish food intake, child dietary diversity, and child diarrheal morbidity. Other outcomes include awareness of the social marketing campaign and knowledge transfer. Primary and secondary fisheries outcomes will be longitudinal difference-in-difference analyses in fisheries yield of mature fish and fisher income and earnings.

The *Samaki Salama* intervention precludes randomization due to the high risk of spill-over effects and the limited number of trap fishers with children under the age of 5. Thus, for the impact assessment, we will conduct a matched intervention/control design to minimize selection bias and test effectiveness on nutrition and fisheries production. Steps have been taken to reduce the risk of selection bias that might arise from the matched design. A pilot study and additional formative research will be used to identify and match communities on important characteristics [socioeconomic status (SES), child nutrition, livelihoods, etc.]. This preliminary data provides additional information needed for external validity and extrapolation of findings to other small fisher households in Kenya and internationally. The longitudinal difference-in-difference design further contributes to the internal validity, accounting for residual confounding, and increases the statistical power to detect intervention effects.

### Sample size

Sample size calculations for the cluster design applied a mean −1.3 height-for-age (HAZ) for the coast region (20) and a hypothesized 0.20 effect size (21). Thus, we estimate requiring a total sample size of 400 households, which includes ~4 clusters per arm (four villages per BMU) and 50–75 households per cluster – assuming 20% losses-to-follow-up (α = 0.05 and 1-β = 0.80). Although this is small number of clusters, we will apply matching techniques a priori and during data analyses (e.g., propensity score matching) to better ensure internal validity.

### Setting

**Figure 3:**
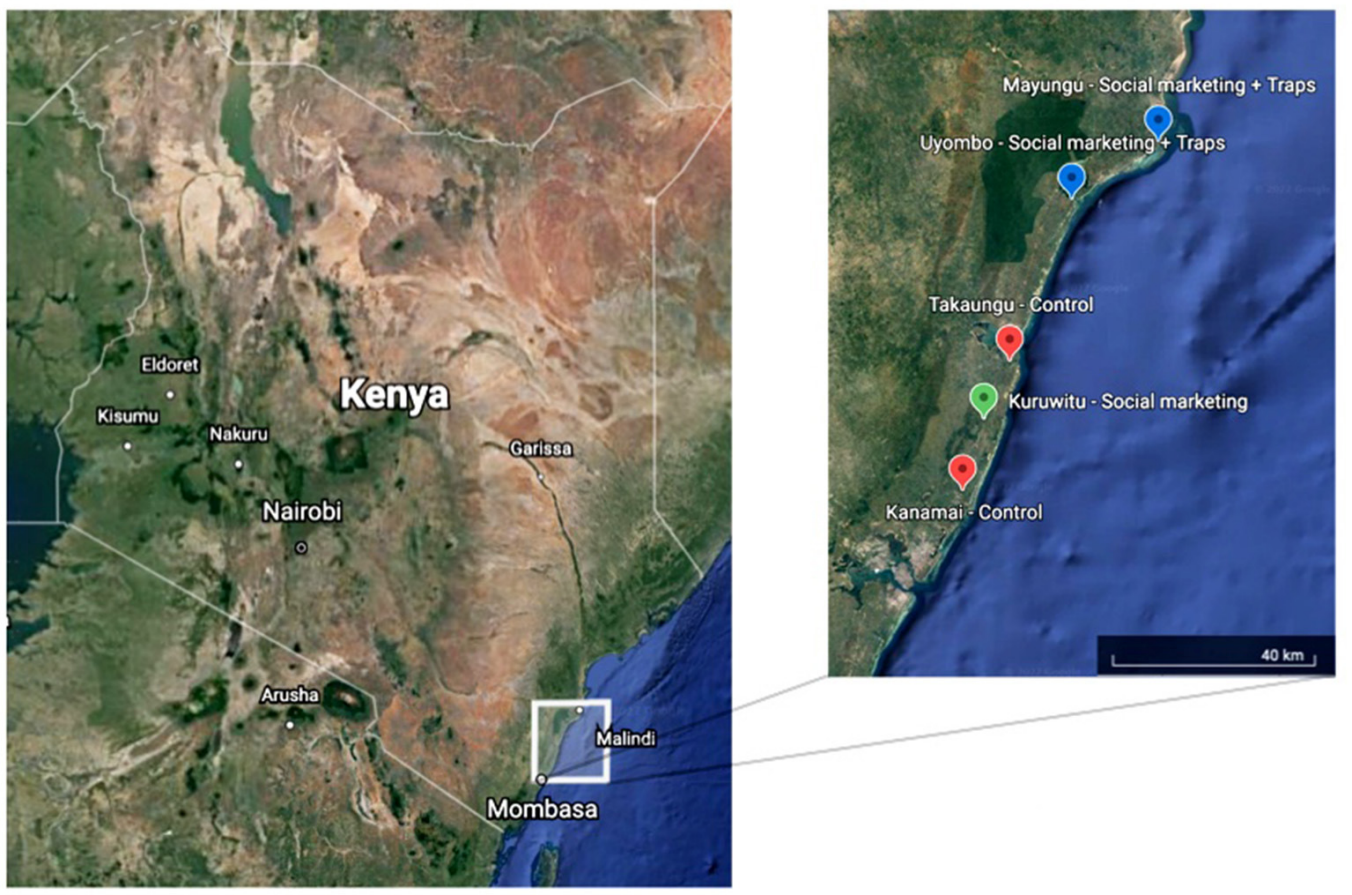
Study area (Source: Google Earth).

The study will be carried out in five distinct areas in Kilifi County, Kenya: Mayungu, Uyombo, Takaungu, Kuruwitu, and Kanamai (Figure 3). Study sites were chosen based on established relationships with the research team, receptivity to the proposed intervention, and proximity and access to marine resources. Kilifi County covers an area of 12,370 km^2^ with a population of 1.45 million and average household size of 4.8 persons (22, 23). The population primarily relies on small-scale fishing, farming activities (raising livestock, tree-cropping, and food-crop production), tourism, and migration to urban centers for their livelihoods (22). Close to half the population lives in poverty (46.4%) and the stunting prevalence (39%) is nearly double the national average, indicating high levels of malnutrition (20, 24). Education levels are low, and the populations are marginalized with limited formal rights to the marine resources on which many of their livelihoods depend.

### Participant eligibility, recruitment, and retention

At the outset of the study, we will identify comparable communities associated with BMUs on the coast of Kilifi County using potential confounding variables: proximity to shoreline or ability to participate in the fishery; presence of no-take fisheries closures; composition of fishing gears used; background nutritional status; usual diets; income and assets; and access to market information. We will draw on existing relationships and data from communities where formative research was previously conducted by the project team. BMU leaders and other stakeholders will be convened to first inform them of the potential project and solicit permission to act in partnership with BMUs to conduct the research.

To be eligible to participate in the study, an individual must meet all of the following criteria:

1. A household member works in small-scale fisheries (self-employed fishers).
2. At least one child in the household aged 6–60 months.
3. Provision of signed and dated informed consent form.
4. For children, informed assent and parental informed consent to participate in the study.
5. Stated willingness to comply with all study procedures and availability for the duration of the study.

An individual who meets any of the following criteria will be excluded from participation in the study:

1. No household member works in small-scale fisheries (self-employed fishers).
2. No child in the household aged 6–60 months.
3. Declines to sign the informed consent.
4. Index child is severely malnourished.
5. Lives outside the study area.
6. Participation in another nutrition or fisheries intervention study within April 2021 - June 2022.

We anticipate a total sample size of 400 small-scale fisher household units (mother, father, and child 6–60 months). There will be 100 in each intervention group (social marketing and social marketing plus modified traps) and 200 in the control group. Table 2 provides a summary of the total number of individuals and anticipated demographics.

**Table 2:** Sample size and demographics.

**Group 1: Control (n = 200)**			**Group 2: Social marketing (n = 100)**			**Group 3: Social marketing + modified traps (n = 100)**		
**Group**	**# of individuals**	**Anticipated sex**	**Group**	**# of individuals**	**Anticipated sex**	**Group**	**# of individuals**	**Anticipated sex**
**Total sample size: 400 total households**								
Mother/caregiver	200	Female	Mother/caregiver	100	Female	Mother/caregiver	100	Female
Fisher	200	male	Fisher	100	Male	Fisher	100	Male
Children 6–60 months	Minimum of 200	Mixed	Children 6–60 months	Minimum of 100	Mixed	Children 6–60 months	Minimum of 100	Mixed

Participant recruitment and enrollment will be conducted by the two Kenyan partner universities (Egerton University and Pwani University). Pwani University, located in Kilifi County and on the coast, is well-positioned to be in continuous interaction with community stakeholders. The Kenya P.I. from Pwani has established relationships in Kilifi over many years of working there and teaches many university students from the neighboring fishing villages. Additionally, the US Co-P.I. from the University of Rhode Island (URI) has worked in this area for over 10 years conducting research on coral reef fisheries management and has long-term working relationships with fishers and BMU leaders. The Kenya Co-P.I. from Egerton University was involved in formative research along the coast and has made contacts with local health care workers and clinics for collecting data and measuring nutrition outcomes.

Recruitment efforts will begin with consultations with key community stakeholders including BMU leaders and board representatives; health care workers in local clinics; community health workers (CHVs); religious leaders; municipal administrators. With their agreement, communities will be matched based on the closest set of characteristics and assigned to control and intervention groups.

Retention efforts will be primarily carried out in tandem with the social marketing campaign. Activities to increase participant engagement – both in control and intervention groups – will include meetings, social gatherings, and project materials (e.g. T-shirts, flyers, lifejackets). These efforts are modeled from the Lulun Project in Ecuador (25) and adapted to the Kenyan context should help retain participants.

### Interventions

The 12-month *Samaki Salama* intervention introduces practices and technologies that build on existing community assets and expressed needs and preferences of small-scale fishers and their households. The first component of the intervention package, nutrition social marketing, is a novel approach to increase nutrition awareness across multiple stakeholders that draws on psychology, marketing, and communications disciplines (25). Distinct from conventional nutrition education interventions that tend to use more didactic approaches and standardized materials, this strategy draws heavily on contextual factors to identify key messages and delivery platforms. Nutrition-focused social marketing campaigns have been shown to promote active engagement of participants throughout the trial, high compliance to the intervention, sustained behavior change, and low losses to follow-up (26). The effectiveness of social marketing increases if targeted messages are repeated and delivered across different platforms (27) and previous studies indicate that 6 months of animal source food nutrition messaging may not be enough to sustain impact (28). Therefore, we propose a 12-month intervention period.

The nutrition social marketing approach will be developed in close collaboration with the social and behavioral change group at USAID Advancing Nutrition. The focus is on promoting four priority behaviors among infants, young children and women of reproductive age in SSF households: (1) caregivers feed fish to young children 6 months to 5 years daily; (2) caregivers feed an age-appropriate diverse diet, including fish, to children 6 months – 5 years daily; (3) caregivers wash hands and the child's hands with soap or ash before feeding; (4) fathers reserve and take home a small portion of fish for child each day. The social marketing plan and audience analysis, including all materials, messaging, and delivery platform relies on formative research conducted in the study area and piloting of materials prior to implementation. As shown in Table 3, messaging centered on the four priority behaviors will be delivered through diverse channels to mothers/caregivers, fathers, and leaders/members of local institutions. The plans to engage these participants groups reflects a multi-level approach to support improved feeding and care behaviors through individual, family, and community level change.

**Table 3:** Social marketing campaign activities and materials.

**Activity**	**Delivered by**	**Branded products**
**Mothers/caregivers**		
Personalized home visits	Staff (Home visitors); CHV	Reminder poster on home actions Letter to fathers about home action Calendar with child growth visual T-shirts Khanga cloth Story book
Cooking demonstrations	Staff; CHVs	Menu game about nutrition foods for children
**Fathers/fishers**		
Workshop series at fish landing sites	Staff	Commitment letter for fishing and home actions T-shirts Stickers Life jackets
**Institutions**		
BMUs orientations	Staff	Banners Posters Stickers
Health worker orientations	Staff, CHVs	Posters Stickers Bags

*Home visits* are a key component of the social marketing campaign and will occur at a three time points (3, 6, and 9 mo.) in both intervention groups. All homes in the intervention groups (*n* = 200) will be visited at each time point. Visits every 3 months will allow the field team enough time to complete one round of visits before starting the next round. Three visits per household is the maximum the field team anticipates being able to complete within the 12-month intervention. During visits, the team's nutrition education specialists will actively engage with the caregiver and child/children to build a positive relationship, understand their individual needs, and foster change. The suite of social marketing materials available to the field team will be the same for all intervention households but we anticipate that the exact information and materials provided will be tailored to meet the needs of the individual household and child/caregiver dyad. The nutrition team will discuss and identify illnesses before introducing age and stage-specific child feeding and hygiene habits (or recommendations) and help caregivers identify next steps and agree on actions geared toward improving feeding and hygiene practices and better health care. The overarching message caregivers will receive is that fish can be a critical source of nutrients for their child and paying special attention to their child's growth and development now has life-long benefits. Discussions, agreements, and actions taken will be documented and tracked by the nutrition team. Local CHVs in both intervention and control communities will be trained to conduct the home visits so caregivers will have a reliable source of information and support even after the project is completed.

Fifteen *cooking demonstrations* will be conducted in intervention communities with an anticipated 10–15 caregiver participants and their children per demonstration. The demonstrations will provide an overview of key nutrition concepts, important nutrients found in fish, and different approaches for fish preparation. Participants will be asked to plan a meal and snacks for their children using a “star foods” menu game and work together to prepare a fish centered meal. Questions will be asked at the beginning and end of the demonstration to better understand participants' nutrition knowledge, what they gained from the demonstration, and feedback for improving future demonstrations. Demonstrations will be conducted in local community spaces identified by the field team.

The project nutrition team will work together with the fisheries team to conduct a series of 10 *fisher workshops* at local BMU offices with fishers in both intervention groups. Table 4 provides a summary of the themes and topics that will be covered at each workshop.

**Table 4:** Themes and topics of fisher workshops.

**Workshop theme**	**Intervention group**	**Discussion topics**
“We provide for our families”	Social marketing + modified traps	What it means to fishers to provide for their families Challenges they face providing for their families Benefits of using modified traps for income, their families nutrition, and fishery sustainability Commitment to using modified traps
	Social marketing	What it means to provide for their families Challenges they face providing for their families Stages of fish growth Activity to estimate mature size of common fish Reasons to focus on catching more mature fish Commitment to catch more mature fish
“We protect our families and communities”	Social marketing + modified traps	Check in on use of modified traps Hopes they have for their children Actions they can take to protect their children's health Encouraging each other to protect their children's nutrition Commitment to bring home some of their catch for their children
“We bring our BMU community”	Social marketing + modified traps	Check in on use of modified traps Refresh knowledge of child nutrition Discuss changes they have noticed in the areas where they fish (environment, fish quantity, fish types etc.) Importance of sharing the information they have received with other fishers Activity to develop a persuasive argument to protect the places where they fish and their children's nutrition Commitment to share what they have learned with new fishers and friends

Fisher training, gear modification, and fishing trap distribution constitute the second piece of the intervention. The use of fishing gears modified to decrease juvenile catch has been shown to lead to greater catch diversity and improve the economic value of fishes (16). Second, fishers can gain a competitive advantage when using new gears by fishing new habitats to catch previously targeted species in novel ways (15). This can reduce the impact of fishing on the environment, and in the case of coastal Kenya, coral reefs. Last, gear modifications have been shown to improve harvest efficiency and promote sustainable fish populations by selecting for mature individuals while at the same time improving fisher revenue (29). Using these rationales, our intervention targets fishers using basket traps and provides them with traps modified with escape gaps so immature fish can escape. Trap distribution will occur at landing sites utilized by participating fishers and at local BMU offices. The fishers will also receive training on how to properly maintain the traps they receive. We hypothesize this type of intervention will reduce fishers' dependence on immature fishes, as well as buffer them from potential market variability (30) and enable them to be more resilient to environmental change (31).

### Data collection and management

Data collection will be conducted by the research staff at the study site under the supervision of the Kenyan investigators. A household and fisher survey will be used to gather data on demographics, socioeconomics, hygiene, sanitation, mother and child health, diet, anthropometry, COVID livelihood impacts, household decision making, awareness/knowledge of fish consumption, and fishing characteristics at baseline and endline. Dietary intakes will be measured using a Kenya-specific semi-quantitative food frequency questionnaire (FFQ) (32). The survey will be developed using the Research Electronic Data Capture (REDCap™) platform and collected electronically using password protected tablets. To assure the quality of data entry the survey will utilize REDCap™'s built-in data validation. The team will also be able to track access of data, instruments, and reports through an electronic audit trail. To minimize missing data the field team coordinators will review all REDCap™ records for completeness prior to uploading the data to the secure REDCap™ server hosted by Washington University in St Louis. If any issues are found the coordinators will follow up with the enumerator responsible for the data entry. Fisheries catch, take home amount, and income data will be collected at least four times monthly at landing sites using paper forms that are then entered into Microsoft Excel by the fisheries field team and stored on password protected computers. Excel data entry is mediated with built-in validation to a given list of marine fish species found locally. All paper forms will be stored in a locked filing cabinet when not being processed. Data will be screened for completeness and consistency on a bi-weekly basis, with archival data stored in the password-protected Box cloud storage platform, and if any issues arise, the research team will follow up with the data collectors.

A process evaluation will be used to monitor and ensure consistent administration of the intervention (fidelity of delivery), adoption, and sustainability, three key implementation outcomes (33). The primary focus is on documenting the transition from inputs [nutrition social marketing and fisher support (modified traps)] to the anticipated outputs, outcomes, and impact (Figure 1). Systematically tracking, documenting, and assessing this part of the impact pathway will allow for a more nuanced understanding of the implementation process, how and why the intervention does/does not have the anticipated impact, and facilitate future replication. Mixed methods will be used to collect information along the impact pathways. Table 6 provides an overview of the implementation process outcomes, methods, and data types.

All data collected for this study will be stored on the REDCap™ platform and on Box, a secure, Health Insurance Portability and Accountability Act (HIPAA) and Family Educational Rights and Privacy Act (FERPA) compliant data storage and sharing online platform.

### Study measures

To evaluate intervention impacts, we will assess primary outcomes of child growth along with fish food intakes, and fisheries yield of mature fish. Secondary outcomes of interest, which are also hypothesized to serve as mediating factors between the intervention and primary outcomes, include indicators for child health and diet (dietary diversity, prevalence of diarrhea) and fisheries earnings. Measurement of the targeted nutrition endpoints will occur at two timepoints as part of a household level survey. The household surveys will also collect information on other relevant measures and potentially confounding factors including household expenditures, and household decision-making. Measurement of fisheries focused endpoints will occur at regular intervals over the course of the 12-month intervention at commonly utilized fish landing sites within the study area. Table 5 summarizes measures that correspond with outcomes of interest.

**Table 5:** Study outcomes.

**Construct**	**Methods**	**Indicator**	**Timepoints**
**Primary outcomes**			
Child growth	Anthropometric measures of height and weight	Length-for-age *Z* score (LAZ) Weight-for-age *Z* score (WAZ) Weight-for-length *Z* score (WLZ)	Baseline, 12 mo.
Fish food intakes	24-h intake survey	Fish food intakes in g	Baseline, 12 mo.
Fisheries yield of mature fish	Fish catch survey at landing sites	Monthly catch per unit effort and fish size distribution	5–10 times per month at randomly stratified days over the course of 12 months
**Secondary outcomes**			
Child diet diversity	24-h intake survey	Diet diversity score	Baseline, 12 mo.
Diarrheal morbidity	HH survey, recall by mother during home visits	Reported acute diarrhea in the last 2 weeks	Baseline, 3, 6, 9, and 12 mo.
Fisheries earnings	Fish catch survey at landing sites	Earnings [in Kenyan shillings (Ksh)] per fishing trip	5–10 times per month at randomly stratified days over the course of 12 mo.

#### Primary outcomes

##### Child growth

Anthropometric measures (length/height, weight) will be collected from children and mothers/caregivers at baseline and endline. The Seca Model 874 (Digital) 440 lbs. × 0.1-lb. resolution and the ShorrBoard^®^ stadiometer will be used to collect weight and length measures, respectively. Measures will be converted to weight-for-age *Z* (WAZ), length-for-age *Z* (LAZ)/HAZ, weight. HAZ and WAZ will be generated using World Health Organization (WHO) (34). The Stata Macro available from WHO will be run to derive the indicators using data on child age in months, sex of the child, and child height/weight. Outliers above HAZ/WAZ > 6 or HAZ/WAZ <−6 will be removed.

##### Dietary assessment/fish food intakes

Dietary intakes will be measured using a Kenya-specific semi-quantitative food frequency questionnaire (FFQ) (32). A comprehensive list of foods consumed in Kenya, and specifically along the coast, will be compiled along with ingredients in common dishes. This will be integrated into the survey as an FFQ for 24-h intakes of women of reproductive age, youth, and children ages 6–60 months. Particular attention will be given to fish foods and other animal source foods which will be asked as 24 h and 7-day recalls. Findings from the FFQ will later be converted to the Feed the Future (FTF) indicators of minimum dietary diversity for women and young children. Finally, infant and young child feeding practices (IYCF) practices will be assessed in accordance with the FTF minimum acceptable diet indicator.

##### Fisheries yield of mature fish

Trained field enumerators will record fish catches at landing sites (fishery-dependent data). The field team will ask permission to count and weigh a fisher's catch when they return from fishing. Upon consent, the enumerators will identify the fish species or genus level and measure the lengths of a sub-sample of the individuals (*n* = 20). Sampling will occur at least four times per month at randomly stratified days, as determined by the moon phase and considering dominant gear types. Monthly catch per unit effort (CPUE) will be determined as the mean daily catch multiplied by the fishing days per month. We will also evaluate species-specific length-frequency distributions to determine the yield of mature individuals. Sustainable yields will be determined by comparing the initial yields vs. the rate of change of yields for each landing site or BMU, based on average length of catch for given species.

#### Secondary outcomes

##### Child diet diversity

The Child Dietary Diversity Score (CDDS) will be calculated using the total number of food groups reported in the food frequency intake portion of the survey. We will use WHO defined food groups: (1) grains, roots, and tubers; (2) legumes and nuts; (3) dairy products (milk, yogurt, cheese); (4) flesh foods (meat, fish, poultry, and liver/organ meats); (5) eggs; (6) vitamin A rich fruits and vegetables; and (7) other fruits and vegetables. If new indicator guidelines are released before data analysis activities of this project are undertaken, we will apply the new definition.

*Diarrheal morbidity* will be calculated using a standard 2-week recall conducted during the household survey and home visits. Questions will assess diarrheal severity including the frequency of diarrhea in the children, presence of blood or fever, use of antimicrobials, and requirement for additional medical care at a clinic or local provider. This data will be used to estimate indicators for acute diarrhea (3 or more liquid or semi-liquid stools in a 24-h period over the last 2 weeks) and persistent diarrhea (lasts 14 days or longer).

##### Fisher revenue

During fisheries yield data collection, enumerators will also ask fishers about their operational costs and the revenue generated from selling the fish. Fisher revenue will be represented as Kenyan shillings (Ksh) per fishing trip. These questions will be informed by cultural norms and objects such as food and equipment used as currency when Kenyan shillings cannot be estimated (e.g., bags of rice). Comparisons will be made at the landing site or BMU level to measure the change in earnings pre- and post-intervention as described by Wamukota et al. (35).

A range of other variables will be assessed to control for cofounding factors associated with the cluster design. These variables include but are not limited to child illness (a standard 2-week recall on infectious illnesses including cough, rhinorrhea, fever, and rash), household consumption and assets, and household decision making [Likert scale instrument that captures common domains of decision-making including purchasing decisions; decisions regarding service use (health, education); decisions regarding children's diet, health and education].

#### Process evaluation

As shown in Table 6, the process evaluation will examine three key implementation outcomes: fidelity, adoption, and sustainability. The *fidelity* outcome will capture the degree to which the intervention was implemented as described in the study protocol, adherence over the course of the intervention, and the quality of program delivery (33). Methods for collecting and documenting implementation fidelity include activity/event counts, semi-structured interviews with intervention participants, reports from the field team, and a baseline/endline survey of caregivers and fishers that assess awareness and knowledge transfer associated with the social marketing campaign.

**Table 6:** Process evaluation outcomes.

**Outcome**	**Methods**	**Data collected**	**Timepoints**
Fidelity	Counts of activities/events Activity/event sign-in sheets	Age of the participant/s, sex (M/F), date, material that was distributed	Rolling basis as activities/events are conducted
	15–20 semi-structured interviews with caregivers and fishers	Project materials viewed Messages that they are aware of Perceptions of the materials and messages Challenges understanding the messages What worked well, what could be improved	6–9 mo.
	Semi-structured discussions pre/post activities	What was learned What worked well, what could be improved	6–9 mo.
	Field team reports	Reports detailing implementation of activities	6 mo., 12 mo.
	Household/fisher survey	Awareness of nutrition messaging	Baseline, 12 mo.
Adoption	Caregiver home visits	Intentions to try, actions since last visit, observations	3, 6, and 9 mo.
	Household/fisher survey	Knowledge transfer linked to the social marketing campaign	Baseline, 12 mo.
	Meetings with fishers	Self-reported use of traps, intentions to try	6–12 mo.
Sustainability	15–20 semi-structured interviews with key informants	What they know about the intervention Perceptions, feasibility for maintaining it once the intervention ends	12 mo.

*Adoption* will focus on better understanding participants intention to try to actualize the information they receive (33). During home visits with caregivers the research team will observe what changes the mother/caregiver has made and their intention to try to act on the messaging in the future. Meetings with fishers will gather similar information as well as asking fishers to report on their use of the modified traps.

The *sustainability* outcome is intended as an initial assessment of local institutions interest and ability to maintain the intervention once it has been completed. Semi-structured interviews with BMU officials, heads of local health clinics, CHVs, and other relevant local government representatives will be used to assess what they know about the intervention, their perceptions of it, and institutions interest and potential for maintaining.

### Analysis

#### Primary and secondary outcomes

Generalized linear regression modeling (GLM), allowing for non-normal distributions, will test the continuous outcomes of HAZ, WAZ, child dietary fish intake, child dietary diversity score, fisheries yield, and fish earnings. As a difference-in-difference analyses, change variables for each outcome will be examined (difference between baseline and endline). For the binomial outcomes of diarrhea morbidity (and other outcomes of stunting and underweight), we will estimate prevalence ratios by the GLM modeling with robust Poisson. If stunting prevalence in this population exceeds the acceptable threshold for use of odds ratios (0.2105), prevalence ratios (PRs) will be used to examine the intervention effect and were considered analogous to relative risk in this longitudinal study. The robust Poisson, with a classic sandwich estimator to correct the inflated variance of standard Poisson, is less affected by outliers.

To test for intervention effectiveness, the two intervention groups will be combined for all hypotheses except the secondary outcome of increased fisher earnings and exploratory hypothesis for differences between social marketing and social marketing + traps intervention groups. Regression models will be adjusted for potential confounding factors including age, sex of the child, corresponding baseline measures, and others found to differ significantly between the trial groups (e.g., maternal education). For fisheries yield, confounding factors will be used to adjust regression models, such as: water temperature, fishing ground area, coral cover, and seasonality. The *P* significance value for Type I error (and one-tailed test) will be *P* < 0.05 and corresponding 95% confidence interval. Diagnostics for regression model assumptions, structure and observations will be applied, and corrective procedures applied as necessary. If selection bias is widely detected with important differences across intervention and control communities, we will apply propensity score analyses (36). Data analyses will be performed with Stata software (version 16.0; StataCorp, College Station, TX) and R (4.1.2).

#### Sub-group analysis

We plan to conduct sub-group analyses for both the primary and secondary endpoints based on child age (6–24, 25–48, and 49–60 mo.) and baseline anthropometry (HAZ/WAZ <−2 and HAZ/WAZ > −2). The justification for this is based on the evidence showing that these characteristics may influence the response effect. Younger children growing more rapidly may show greater response in HAZ. As well, children stunted at baseline may also show a greater response to the intervention.

#### Process evaluation

A range of approaches will be used to analyze the data collected as part of the process evaluation. NVivo software will be used to code and analyze the qualitative data from the semi-structured interviews, discussions, home visit notes, and observations. A directed content analysis approach will be used with two rounds of coding (37). Phase one of the process will be closed coding using a codebook developed from the interview guides. A second round of open coding will be used to clarify any of the new ideas that were identified in phase one. Once open coding has been completed, code mapping will be conducted, and codes will be grouped into hierarchies to organize evidence as themes emerge. Throughout this process, the research team will document reflections on the content of the interviews. This documentation along with the notes of the field research coordinator will be analyzed to capture insights and possible lines of additional inquiry. Counts of actual activities and events will be compared with the project workplan to assess implementation fidelity and coverage. Differences between baseline and endline awareness and knowledge transfer captured in the household/fisher survey will be analyzed using R (4.1.2). An anticipated output of the analysis is a paper that details the implementation process, challenges that were faced, successes, and lessons learned for future replication.

## Discussion

This paper describes the study protocol for *Samaki Salama*, a three-arm longitudinal matched cluster study which tests the effectiveness of an integrated intervention to address malnutrition and its intersections with nutrition security and fisheries sustainability in Kilifi County, Kenya.

Our study offers an exciting opportunity to contribute to the nutrition-sensitive intervention evidence base. This “research for development” project can serve as a model for other programs in Kenya and globally for support of sustainable small-scale fisheries production and food security. Strategically positioned in Kenya, it offers new prototypes of support to entire small fisher households, marine biodiversity-nutrition linkages, and the application of scalable technologies. The escalating negative impact of climate change on the health and livelihoods of small-scale producers amplifies the need for this type of integrated approach and programming.

There is a growing body of literature illustrating the effectiveness of nutrition-focused social marketing campaigns to promote active engagement of participants, high compliance to the intervention, sustained behavior change, and low losses to follow-up. This study is designed to build on this literature and expand it to social-ecological contexts where there has been little to no application.

Developing and implementing a study during the COVID-19 pandemic adds an additional layer of challenges to any type of applied research. As part of the process evaluation the research team will methodically document the implementation process to facilitate replication and translation to similar SSF systems and clearly convey study adaptations necessitated by the pandemic.

Many well-intentioned interventions fall short on delivering their intended impact. Due to a lack of transparent systematic mixed methods evaluations the reason/s for limited success are often unclear and mistakes are repeated. This study can serve as an example of design, implementation, and evaluation approaches that offer clear lessons learned for future interventions and facilitate sustained change.

## Ethics statement

The studies involving human participants were reviewed and approved by the Human Resource Protection Office of Washington University in St. Louis (# 202101019) and the Pwani University Ethics Review Committee (#ERC/EXT/003/2020R). Written informed consent to participate in this study was provided by the participants' legal guardian/next of kin.

## Author contributions

LI, AW, EK-M, MC, and AH conceptualized the study. IB and LI wrote the manuscript with support from AW, EK-M, AH, CL, RC, MKC, and LS. IB and CL designed the process evaluation. LI and EK-M developed the nutrition methods and statistical analysis plan. AW and AH developed the fisheries methods. IB, RC, CS, FM, CC, and JC developed the data collection and management systems for the study. LS developed the materials and plan for the social marketing campaign. AW, EK-M, CS, FM, CO, CC, and JC provided technical guidance for implementing the study in Kenya. All authors participated in the drafting of this manuscript and have read and approved the final manuscript.

## Funding

This project and publication are made possible by the generous support of the American people provided by the Feed the Future Innovation Lab for Fish through the United States Agency for International Development (USAID). The Feed the Future Innovation Lab for Fish is managed by Mississippi State University through an award from USAID (Award No. 7200AA18CA00030; M. Lawrence, PI) and provides support to these projects: Grant No. 193900.312455.02; LI, PI, Grant No.193900.312455.04; AH, co-PI, Grant No.193900.312455.18; EK-M, co-PI, Grant No.193900.312455.17B; AW, co-PI. The funder has no role in the design and implementation of this study.

## Conflict of interest

Author LS was employed by JSI Research & Training Institute, Inc. The remaining authors declare that the research was conducted in the absence of any commercial or financial relationships that could be construed as a potential conflict of interest.

## Publisher's note

All claims expressed in this article are solely those of the authors and do not necessarily represent those of their affiliated organizations, or those of the publisher, the editors and the reviewers. Any product that may be evaluated in this article, or claim that may be made by its manufacturer, is not guaranteed or endorsed by the publisher.

## Author disclaimer

The contents are the responsibility of the authors and do not necessarily reflect the views of USAID or the United States Government.

## References

[1] Black RE Victora CG Walker SP Bhutta ZA Christian P De Onis M . Maternal and child undernutrition and overweight in low-income and middle-income countries. Lancet. (2013) 382:427–451. 10.1016/S0140-6736(13)60937-X 23746772

[2] Iannotti LL Blackmore I Cohn R Chen F Gyimah EA Chapnick M . Aquatic animal foods for nutrition security and child health. Food Nutr Bull. (2022) 43:127–47. 10.1177/03795721211061924 34905969

[3] FAO. The State of Food Security and Nutrition in the World 2020. Transforming Food Systems for Affordable Healthy Diets. Rome, Italy (2020). Available online at: https://reliefweb.int/report/world/state-food-security-and-nutrition-world-2020-transforming-food-systems-affordable (accessed December 20, 2020).

[4] Willett W Rockström J Loken B Springmann M Lang T Vermeulen S . Food in the anthropocene : the EAT – Lancet Commission on healthy diets from sustainable food systems. Lancet. (2019) 393:447–92. 10.1016/S0140-6736(18)31788-4 30660336

[5] Béné C Arthur R Norbury H Allison EH Beveridge M Bush S . Contribution of fisheries and aquaculture to food security and poverty reduction: assessing the current evidence. World Dev. (2016) 79:177–96. 10.1016/j.worlddev.2015.11.007

[6] UNEP. Smallholders, Food Security and the Environment. Rome, Italy (2013).

[7] World Bank Food and Agriculture Organization WorldFish. Hidden Harvests: The Global Contribution of Capture Fisheries, Economic and Sector Work Report No. 66469-GLB. Washington DC (2012).

[8] Bennett NJ Govan H Satterfield T. Ocean grabbing. Mar Policy. (2015) 57:61–8. 10.1016/j.marpol.2015.03.026

[9] Bavinck M Berkes F Charles A Dias ACE Doubleday N Nayak P . The impact of coastal grabbing on community conservation–a global reconnaissance. Marit Stud. (2017) 16:1–17. 10.1186/s40152-017-0062-8

[10] Cohen PJ Allison EH Andrew NL Cinner J Evans LS Fabinyi M . Securing a just space for small-scale fisheries in the blue economy. Front Mar Sci. (2019) 6:1–8. 10.3389/fmars.2019.00171

[11] Worm B Barbier EB Beaumont N Duffy JE Folke C Halpern BS . Impacts of biodiversity loss on ocean ecosystem services. Science. (2006) 314:787–91. 10.1126/science.1132294 17082450

[12] McCauley DJ Pinsky ML Palumbi SR Estes JA Joyce FH Warner RR. Marine defaunation: animal loss in the global ocean. Science. (2015) 347:1255641. 10.1126/science.1255641 25593191

[13] IPBES. Global Assessment Report on Biodiversity and Ecosystem Services of the Intergovernmental Science- Policy Platform on Biodiversity and Ecosystem Services. Brondizio ES Settele J Díaz S Ngo HT editors. Bonn: IPBES Secretariat (2019).

[14] Kolding J Bene C Bavinck M. Small-scale fisheries - importance, vulnerability, and deficient knowledge. In:Garcia S Rice J Charles A, editors. Governance for Marine Fisheries and Biodiversity Conservation: Interaction and Co-evolution. Hoboken, NJ: Wiley-Blackwell (2014), p. 317–32. 10.1002/9781118392607.ch22

[15] Selgrath JC Gergel SE Vincent A. Shifting gears: diversification, intensification, and effort increases in small-scale fisheries. PLoS ONE. (2018) 13:e0190232. 10.1371/journal.pone.0190232 29538370 PMC5851533

[16] McClanahan TR Hicks C Darling E. Malthusian overfishing and efforts to overcome it on Kenyan coral reefs. Ecol Appl. (2008) 18:1516–29. 10.1890/07-0876.1 18767626

[17] Samoilys M Osuka K Maina GW Obura D. Artisanal fisheries on Kenyas to overcome it on Kenyan cor reveal management needs. Fish Res. (2017) 186:177–91. 10.1016/j.fishres.2016.07.025

[18] Njiru M Nzungi P Getabu A Wakwabi E Othina A Jembe T . Are fisheries management, measures in Lake Victoria successful? The case of Nile perch and Nile tilapia fishery. Afr J Ecol. (2007) 45:315–23. 10.1111/j.1365-2028.2006.00712.x

[19] McClanahan TR. Marine reserve more sustainable than gear restriction in maintaining long-term coral reef fisheries yields. Mar Policy. (2021) 128:1–9. 10.1016/j.marpol.2021.104478

[20] Kenya DHS. Kenya National Bureau of Statistics, Ministry of Health, the National AIDS Control Council, the National Council for Population and Development, Kenya. (2014).

[21] Bhutta ZA Das JK Rizvi A Gaffey MF Walker N Horton S . Evidence-based interventions for improvement of maternal and child nutrition: what can be done and at what cost? Lancet. (2013) 382:452–77. 10.1016/S0140-6736(13)60996-4 23746776

[22] County Government of Kilifi. Integrated Development Plan 2018-2022: Towards Realizing People-Focused Transformation for Wealth Creation. Kilifi (2018).

[23] Kenya National Bureau of Statistics. 2019 Kenya Population and Housing Census Volume 1: Population by County and Sub-County. (2019). Available online at: https://www.knbs.or.ke/?wpdmpro=2019-kenya-population-and-housing-census-volume-i-population-by-county-and-sub-county (accessed February 5, 2020).

[24] Kenya Institute for Public Policy Research and Analysis. Kenya Economic Report 2020: Creating an Enabling Environment for Inclusive Growth in Kenya. Nairobi, Kenya (2020).

[25] Gallegos-Riofrío C Waters W Salvador J Carrasco A Lutter C Stewart C . The Lulun Project's social marketing strategy in a trial to introduce eggs during complementary feeding in Ecuador. Matern Child Nutr. (2018) 14(Suppl 3):e12700. 10.1111/mcn.12700 30332535 PMC6865975

[26] Iannotti L Lutter CK Stewart CP Gallegos Riofrn a trial to introduce eg . Eggs in early complementary feeding and child growth: a randomized controlled trial. Pediatrics. (2017) 140:e20163459. 10.1542/peds.2016-3459 28588101

[27] Mangold WG Faulds DJ. Social media: the new hybrid element of the promotion mix. Bus Horiz. (2009) 52:357–65. 10.1016/j.bushor.2009.03.002

[28] Iannotti L Chapnick M Nicholas J Gallegos-Riofrio C Moreno P Douglas K . Egg intervention effect on linear growth no longer present after two years. Matern Child Nutr. (2020) 16:e12925. 10.1111/mcn.12925 31849201 PMC7083396

[29] Gomes I Erzini K McClanahan TR. Trap modification opens new gates to achieve sustainable coral reef fisheries. Aquat Conserv Mar Freshw Ecosyst. (2014) 24:680–95. 10.1002/aqc.2389

[30] Allison EH Horemans B. Putting the principles of the sustainable livelihoods approach into fisheries development policy and practice. Mar Policy. (2006) 30:757–66. 10.1016/j.marpol.2006.02.001

[31] Cinner JE Huchery C Darling ES Humphries AT Graham NA Hicks CC . Evaluating social and ecological vulnerability of coral reef fisheries to climate change. PLoS ONE. (2013) 8:e74321. 10.1371/journal.pone.0074321 24040228 PMC3770588

[32] Hu FB Rimm E Smith-Warner SA Feskanich D Stampfer MJ Ascherio A . Reproducibility and validity of dietary patterns assessed with a food-frequency questionnaire. Am J Clin Nutr. (1999) 69:243–9. 10.1093/ajcn/69.2.243 9989687

[33] Proctor E Silmere H Raghavan R Hovmand P Aarons G Bunger A . Outcomes for implementation research: conceptual distinctions, measurement challenges, and research Agenda. Adm Policy Ment Heal Ment Heal Serv Res. (2011) 38:65–76. 10.1007/s10488-010-0319-7 20957426 PMC3068522

[34] WHO Multicentre Growth Reference Study Group. WHO Child Growth Standards based on length/height, weight and age. Acta Paediatr Suppl. (2006) 450:76–85. 10.1111/j.1651-2227.2006.tb02378.x 16817681

[35] Wamukota A Brewer TD Crona B. Market integration and its relation to income distribution and inequality among fishers and traders: the case of two small-scale Kenyan reef fisheries. Mar Policy. (2014) 48:93–101. 10.1016/j.marpol.2014.03.013

[36] Guo SY Fraser MW. Propensity Score Analysis: Statistical Methods and Applications, 1st ed. Thousand Oaks, CA: Sage Publications (2010).

[37] Miles MB Huberman AM Saldana J. Qualitative Data Analysis A Methods Sourcebook. 3rd ed. Thousand Oaks, CA: Sage Publications (2014), p. 408.

